# Clinical outcome of ultrasound-guided percutaneous microwave ablation on colorectal liver metastases

**DOI:** 10.3892/ol.2014.2106

**Published:** 2014-04-29

**Authors:** JIANBIN WANG, PING LIANG, JIE YU, MING-AN YU, FANGYI LIU, ZHIGANG CHENG, XIAOLING YU

**Affiliations:** Department of Interventional Ultrasound, Chinese PLA General Hospital, Beijing 100853, P.R. China

**Keywords:** microwave ablation, ultrasound-guided, liver metastases, colorectal cancer

## Abstract

The present study aimed to assess the feasibility, safety and efficiency of ultrasound-guided percutaneous microwave ablation (MWA) on liver metastases from colon or rectal cancer. Patients who received MWA therapy for liver metastases from colon or rectal cancer between June 2009 and May 2012 were enrolled in the study. Follow-up data was collected from the patients in order to statistically analyze the adverse effects, concurrent disease and survival status. Of the total 115 patients, 62 presented with colon cancer and 53 with rectal cancer. A total of 78 patients were male and 37 were female. The patient age ranged between 30 and 86 years [mean ± standard deviation (SD), 59.46±11.79 years]. The number of overall ablation lesions was 165, and the diameter of the lesions ranged between 1.3 and 5.0 cm (mean ± SD, 3.10±1.05 cm). Subsequent to treatment, the mean (± SD) hospitalization time was 4.69±2.08 days (range, 2–10 days). The median follow-up time was 28 months (range, 12–48 months) and 5 patients were lost to follow-up. The pain grade was recorded between the 4th and 6th degree following treatment in 23 patients. The body temperatures of 35 patients reached >38°C, with the longest time at this temperature recorded as 5 days. Following treatment, 5 patients presented with pleural effusion and required thoracocentesis and drainage. Following ablation, the rate of local progression was 11.82%. The recurrence rates were 27.8, 48.4 and 59.3% and the cumulative survival rates were 98.1, 87.1 and 78.7% in years 1, 2 and 3 post-treatment, respectively. A total of 14 patients succumbed. No significant differences were observed in the liver metastases of colorectal cancer with regard to gender, age, number of lesions, lesion size and pathological differentiation (P>0.05). Also, no significant difference was observed in the recurrence or cumulative survival rates for years 1, 2 and 3 years post-treatment (P>0.05). In conclusion, ultrasound-guided percutaneous MWA is a safe and competent way to treat inoperable colorectal liver metastases.

## Introduction

Colorectal cancer is a malignancy with high incidence. The main cause of mortality is metastases, and the liver is the most common target organ in colorectal cancer (1). In total, 20–25% of colorectal cancer patients are diagnosed with liver metastases, and another 20–25% with metachronous hepatic metastases. The main method used to treat colorectal liver metastases (CRLM) is hepatectomy, however, only 10–20% patients are suitable for this procedure (2). Image-guided thermal ablation has been developed and is now widely used to treat liver tumors. Additionally, radiofrequency ablation (RFA) is a safe, well-tolerated, repeatable and less invasive approach to treat CRLM (3,4). Whilst, microwave ablation (MWA) is highly valued, as it has a wide ablation diameter, high ablation rates, low heat sink effect and short duration (5,6), the available studies of MWA on CRLM are fewer in number than those of RFA. The present study aimed to assess the feasibility, safety and efficiency of ultrasound-guided percutaneous microwave ablation (MWA) on liver metastases from colon or rectal cancer.

## Materials and methods

### Patients

All patients enrolled in the present study were treated for CRLM using ultrasound-guided percutaneous MWA between June 2009 and May 2012 in the Department of Interventional Ultrasound, in the Chinese People’s Liberation Army General Hospital (Beijing, China). The study was approved by the ethics committee of Chinese PLA General Hospital (Beijing, China).

### Inclusion criteria

To be eligible for MWA, the following inclusion criteria were used: Tumor diameters of <5 cm for single liver tumors; tumor diameters of <3 cm for 2–3 tumors; no invasion of the blood vessels, bile duct and adjacent organs; no distant metastasis; and normal coagulation.

### Treatment

All necessary examinations were performed prior to the treatment, and signed informed consent was obtained from each patient. Liver lesions were clearly displayed upon two-dimensional ultrasound, and if the image was not clear, it was located by contrast-enhanced ultrasound. All the patients were treated with intravenous anesthesia, a cooled-shaft microwave system (KY-2000; Kangyou Medical, Nanjing, Jiangsu, China), two coaxial cables and two water-pumping machines, which could simultaneously drive two 15-gauge polytetrafluorethylene-coated cooled-shaft antennae. The generators were able to produce 1–100 W of power at 2,450 MHz. Three types of antennae, with 0.5, 0.7 and 1.1 cm tips, were chosen according to the size of the tumor, diameter <2, 2–3 and >3 cm, respectively. A thermal monitoring system with 21-gauge thermocouple needles was equipped in the MWA machine. These needles function by monitoring the real-time temperature percutaneously at a specified location (7,8).

### Follow-up visits

Subsequent to 3 days of treatment, all patients were examined by contrast-enhanced ultrasound to determine the inactivated condition of the tumor and to establish whether any supplementary treatment was required. Subsequent to this, changes in the pain grade and temperature, and results of routine blood and liver function tests were observed. The patients were examined by enhanced computed tomography (CT)/magnetic resonance imaging (MRI) or ultrasound imaging in months 1, 3, 6, 9 and 12 post-treatment. A follow-up study was performed every 3–6 months.

## Results

### Baseline data

From a total of 180 patients, 115 were enrolled in the present study according to the inclusion criteria. A total of 62 patients presented with colon cancer and 53 with rectal cancer. Highly-differentiated cancer was detected in 10 patients, moderately-differentiated in 76 and poorly-differentiated in 29. The patients consisted of 78 males and 37 females. The age range was between 30 and 86 years old [mean ± standard deviation (SD), 59.46±11.79]. There were 165 ablation lesions, with diameters ranging between 1.3 and 5.0 cm (mean ± SD, 3.10±1.05 cm); 85 lesions were ablated once and 80 lesions were ablated twice. The mean (± SD) power level used was 50.3±7.2 W (range, 45–60 W) and the mean (± SD) duration of treatment was 5.6±2.7 min (range, 2–8 min). Following treatment, the mean (± SD) hospitalization time was 4.69±2.08 days (range, 2–10 days). According to the location of the liver metastasis, two groups were defined: The colon cancer group and the rectal cancer group.

### Comparison of baseline data between colon and rectal cancer groups

No significant differences were observed between the colon and rectal cancer groups with regard to gender, age, number of lesions, lesion size, pathological differentiation or hospitalization time following ablation (P>0.05; Table I).

### Comparison of recurrence and cumulative survival rates between colon and rectal cancer groups

Up to May 2013, the patients had been followed up for 28 months (median follow-up time; range, 12–48). A total of 5 cases were lost to follow up, with a rate of 4.35%. Subsequent to ablation, 13 cases exhibited intrahepatic lesions, 14 cases exhibited new liver lesions and 23 cases manifested other organ metastases. In total, 14 patients succumbed; 9 due to respiratory failure, 4 due to failure of liver function and 1 due to cerebral hemorrhage. The cumulative recurrence rates were 27.8, 48.4 and 59.3% and the cumulative survival rates were 98.1, 87.1 and 78.7% in years 1, 2 and 3 years post-treatment, respectively. No significant differences were observed between the cumulative recurrence and cumulative survival rates between the two groups (P>0.05) (Table II).

### Major adverse reactions following ablation

Following ablation, adverse reactions were mainly recorded as regional pain, fever and hepatic insufficiency. The pain grade was recorded between the 4th and 6th degree following treatment in 23 patients. The body temperature of 35 patients was >38°C, with the longest period at this temperature recorded as lasting for 5 days. A total of 5 cases required thoracocentesis and drainage for pleural effusions (3 patients with colon cancer and 2 with rectal cancer). No significant difference was observed in the white blood cell count, alanine aminotransferase level, presence of fever, time >38°C or pain grade between the two groups (P>0.05). Additionally, no patients suffered hepatic abscesses, peritonitis, other infections or succumbed to the treatment. The principal adverse reactions of the colon and rectal cancer groups following ablation are shown in Table III.

## Discussion

The disease incidence of colorectal cancer is high, and 40–50% of colorectal cancer patients will develop liver metastases (9). Currently, the main way to treat CRLM is via excision. The 5-year survival rate can reach 30–40%, however, only 20% of patients with liver metastases can be completely cured (10). Patients with unresectable CRLM have previously been shown to live on average for only 8–10 months without treatment, with a 5-year survival rate of <5%. With the application of chemotherapeutics and new target drugs, this survival rate has been shown to be enhanced to 20–24 months, and a few of the CRLM patients in whom it was first believed that excision could not be performed have been found to be able to withstand a resection with curative intent (11,12). However, following chemotherapy, the condition of the patients generally changes. Minimally invasive treatment has become a reasonable choice for the majority of patients who reject liver tumor resection.

In the past two decades, ultrasound-guided minimally invasive treatment has developed so fast that transarterial chemoembolisation (TACE) and thermal ablation therapy have become widely used. TACE is used merely as a palliative therapy when CRLM cannot be excised or the patient rejects surgery (13). However, thermal ablation therapy has become one of the main therapies to cure CRLM, and RFA has been a more recent alternative treatment method. RFA is able to excise a single CRLM (diameter, <3 cm) and has an effect equal to that of direct excision. Moreover, with regard to the small tumors that are difficult to reach and hide in the depths of the liver, RFA, to a certain extent, can treat CRLM better than direct excision (14,15). As is shown by a number of studies of RFA on unresectable CRLM, the 3-year survival rates range between 37 and 77%, while the 5-year rates are between 27 and 36% (16–18). Due to intrahepatic micrometastases and incomplete tumor destruction, the use of RFA is associated with the risk of intrahepatic and local disease recurrence (19). Certain studies of liver recurrence patterns for CRLM following RFA have indicated intrahepatic recurrence rates of between 32 and 62.5% (14,20–22).

Compared with RFA, MWA has the merit of faster local heating and a wider range of ablation zone (23,24). MWA can be used with multiple probes rendering larger tissues down in size in a shorter time. Due to active heating, MWA, unlike RFA, is not affected by charred and desiccated tissue at the tip of the probe, leading to more uniform and reliable tissue ablation zones (25). This study has a short follow-up period and the patients should continue to be followed up until a 5-year survival rate may be calculated.

The principal adverse reactions following ablation are low-grade local pain and fever, which can be relieved following expectant treatment (24). Inflammatory reactions and hepatic insufficiency may be caused by absorption of intrahepatic tumor necrosis, which may be recovered from within 2–7 days. Following ablation, pleural effusion, which is associated with a small distance between ablation lesions and diaphragmatic muscle, can be absorbed automatically by the body or be drained by surgery (26). Noticeably, another concurrent disease, tumor metastasis in the needle tract, should be protected against by way of extraction from the original tract and by heating the tract (27). Neither implantation metastases nor serious infections and mortality are associated with MWA. Unlike surgery, MWA has the advantages of less concurrent diseases, a fast recovery and a higher quality of life.

The limitations of the present study included the fact that the data was only from one center, involved few cases, has a short follow-up time and does not contain a comparison with other therapies. To conclude, although ultrasound-guided percutaneous MWA is a safe and competent way to treat inoperable colorectal cancer in liver metastases, the conclusions require elucidation by a multicenter randomized controlled study.

## Figures and Tables

**Table I:** Comparison of baseline data between colon and rectal cancer groups.

Parameter	Colon (n=62)	Rectal (n=53)	P-value
Gender, n			
Male	38	40	0.155
Female	24	13	
Age, years	59.21±13.06	59.74±10.21	0.806
Number of lesions, n			
1	38	37	0.571
2	18	13	
3	6	3	
Maximum diameter, cm	2.86±0.97	3.1±60.86	0.086
Pathological differentiation, n			
High	5	5	0.593
Moderate	39	37	
Poor	18	11	
Hospitalization time, days	4.73±2.51	4.41±2.69	0.355

**Table II:** Comparison of recurrence rate and mortality rate between colon and rectal cancer groups.

	Recurrence rate			Cumulative survival rate		
		
Group	1-year	2-year	3-year	1-year	2-year	3-year
Colon, %	30.0	48.1	61.1	98.4	88.6	78.7
Rectal, %	36.0	49.7	57.5	97.9	85.8	78.6
P-value	0.441	0.908	0.749	0.546	0.744	0.812

**Table III:** Comparison of adverse reaction between two groups.

Parameter	Colon (n=62)	Rectal (n=53)	P-value
WBC on 1st day, ×10^9^/l	9.36±3.65	8.44±3.62	0.182
ALT on 1st day, U/l	171.45±103.65	195.59±122.18	0.254
Fever			
None	19	15	0.884
<38°C	24	22	
≥38°C	19	16	
Time >38°C, days	0.87±1.38	0.78±1.24	0.748
Pain grade			
0	35	32	0.839
1–3	14	11	
4–6	13	10	

WBC, white blood cells; ALT, alanine aminotransferase.
